# Fluorescence-guided lung nodule identification during minimally invasive lung resections

**DOI:** 10.3389/fsurg.2022.943829

**Published:** 2022-07-18

**Authors:** Riccardo Tajè, Filippo Tommaso Gallina, Daniele Forcella, Giulio Eugenio Vallati, Federico Cappelli, Federico Pierconti, Paolo Visca, Enrico Melis, Francesco Facciolo

**Affiliations:** ^1^ Thoracic Surgery Unit, IRCCS Regina Elena National Cancer Institute, Rome, Italy; ^2^ Radiology Unit, IRCCS Regina Elena National Cancer Institute, Rome, Italy; ^3^ Anesthesiology Unit, IRCCS Regina Elena National Cancer Institute, Rome, Italy; ^4^ Pathology Unit, IRCCS Regina Elena National Cancer Institute, Rome, Italy

**Keywords:** NSCLC, NIR-guided surgery, minimally invasive thoracic surgery, RATS, VATS, lung cancer

## Abstract

In the last few years, minimally invasive surgery has become the standard routine practice to manage lung nodules. Particularly in the case of robotic thoracic surgery, the identification of the lung nodules that do not surface on the visceral pleura could be challenging. Therefore, together with the evolution of surgical instruments to provide the best option in terms of invasiveness, lung nodule localization techniques should be improved to achieve the best outcomes in terms of safety and sensibility. In this review, we aim to overview all principal techniques used to detect the lung nodules that do not present the visceral pleura retraction. We investigate the accuracy of fluorescence guided thoracic surgery in nodule detection and the differences among the most common tracers used.

## Introduction

Along with the diffusion of lung cancer screening, an increasing number of ground glass opacities (GGO) and sub-centimetric pulmonary nodules are detected. Although both GGO and sub-centimetric nodules are non-specific radiological features, a consistent rate of these findings may be revealed as early-stage lung cancer requiring surgical resection (1, 2).

Minimally invasive thoracic surgery is now the new paradigm in the treatment of Non-Small Cells Lung Cancer (NSCLC) (3, 4). Video-thoracoscopic and robotic approaches have demonstrated better perioperative outcomes compared with the open approach. Although controversies still remain with regard to intraoperative lymph node assessment, the long-term oncological outcomes seem to be similar to those of thoracotomy (5). Nonetheless, irrespective of whether surgical techniques are in tune with the rapid technological advancements of the last few decades, nodule detection is still restricted to a visual inspection of indirect signs, such as visceral pleura retraction, and manual palpation (6). However, sub-centimetric nodules or GGO lesions, especially if deeply located in the parenchyma, may let the visceral pleura unaltered, and video-thoracoscopic access or insensitive robotic instruments may hinder a manual inspection of the lung. Nodule localization failure leads to conversion to thoracotomy in up to 63% of the procedures. Nonetheless, a vague nodule localization can result in an insufficient margin, especially in sub-lobar resections (7–9). Thus, several strategies such as hook wire positioning and intraoperative fluoroscopy have been used to pre-operatively localize pulmonary nodules and ensure adequate margin, but some complications have been recorded (10–12).

Fluorescence and near-infrared (NIR) fluorescence guided surgery have been recently implemented in surgical oncology to localize hard-to-detect nodules and to ameliorate surgical radicality (6, 13). This technique takes advantage from direct injection inside or near the targeted nodule of a fluorescent tracer. The tracer can be injected by percutaneous fine needle puncturing under CT guidance or through endobronchial navigation. In the latter, a 3-dimensional virtual reconstruction of the patient lung is obtained based on CT imaging. The airway proximal to the nodule is then identified and the tracer is injected through a bronchoscopic catheter (14).

The primary endpoint of this review is to establish the localization effectiveness of fluorescence-guided thoracic surgery in nodule detection. The secondary endpoints of this study are to evaluate the differences among the most common tracers used for nodule detection, in order to establish the most used tracer dosage, evaluate the radiological characteristics of the detected nodules, nodule diameter, and distance between the nodule and the visceral pleura, establish the best time interval between nodule marking and surgery, and discern the characteristics of the failure of the marking procedure.

## Methods

### Search strategy

The literature search has been done on PubMed. Only English language studies published before March 2022 have been included.

References of the studies found with the PubMed search terms and of previously published literature reviews on the topic have been reviewed as a supplement by two independent reviewers. The study selection process and the terms used in the search are provided in the Supplementary Material in accordance with PRISMA guidelines. For the search strategy, no filters or limits have been applied.

### Inclusion and exclusion criteria

All studies involving fluorescence-guided adult human thoracic surgery have been included. Furthermore, only studies involving more than 10 marking procedures were included. Case reports, reviews, the category how-to-do-it, and technical studies have been excluded. Clinical heterogeneity has been assessed by two independent reviewers. Enrolled studies included consecutive or random samples of patients with pulmonary nodules. Case-control studies have been excluded, unless data on the results of the fluorescence-guided subgroup could be undoubtedly retrieved.

As one of the features analyzed in this study is to assess the NIR dye-administered dosage and pharmacodynamics characteristics of the dye, studies involving patients with age below 18 years old have been excluded. Finally, studies in which data on the used fluorescent dye results are lacking have been excluded.

### Data extraction

From each study, two independent reviewers collected and analyzed in an excel sheet the following data: the demographic characteristics of the patients; the number, dimension, distance from the visceral pleura, and radiological features of the labelled nodules; the type, dosage, administration modality, duration, marking procedure-related complications, and dye tracer-related toxicities of the adopted fluorescent tracer; the time interval between dye tracer administration and surgery; the type and duration of surgical resection.

### Characteristics of the studies

Of the 892 studies identified from PubMed search, 55 studies have been considered eligible for this review (15–69).

## Results

**Figure 1:**
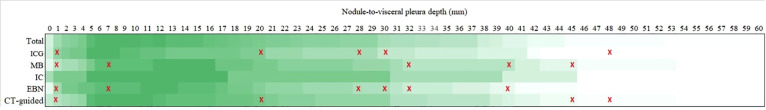
Localization effectiveness of the main techniques according to the depth from the nodule to the visceral pleura.

The results are summarized in Table 1. A total of 3399 patients presenting with 3741 pulmonary nodules were enrolled in this systematic review. Particularly, the average nodule dimension was under 10 mm in 29 studies ranging from 2 to 46 mm. The radiological feature of the nodule revealed a solid morphology in 699 nodules, while 602 nodules were subsolid and 1625 were GGO. Finally, the remaining 12 nodules were cavitary. In 1129 nodules, the radiological appearance of the nodule was not reported. The distance from the nodule to the visceral pleura ranged from 0 to 60 mm. The outcomes of the principal techniques analyzed in this review are summarized in Figure 1.

**Table 1:** Patients and techniques characteristics.

	Total	ICG	MB	IC	Mixed	CT guided	EBN
***N* of articles**	55	18	24	8	5	30	22
***N* of patients**	3399	1213	1533	442	211	2324	1042
***N* of nodules**	3741	1289	1673	538	241	2548	1162
**Mean nodule dimension (*n* of studies)**							
Studies with diameter ≤ 10 mm	29	12	13	4	2	17	11
Studies with diameter > 10 mm	25	6	9	4	4	12	9
**Radiological characteristics (*n* of nodules)**							
Solid	699	145	248	218	88	396	262
Sub-solid	902	286	189	79	48	381	217
GGO	1625	648	657	215	105	1170	383
Other	12	1	4	7	0	3	9
**Mean nodule to visceral pleura distance (*n* of studies)**							
Studies with nodule to pleura ≤ 10 mm	27	12	12	2	1	18	7
Studies with nodule to pleura > 10 mm	18	2	10	3	3	10	7
**Marking procedure (*n* of studies)**							
CT-guided	30	11	16	3	0	30	–
EBN	21	4	8	5	5	–	22
Mixed	3	3	0	0	0	–	–
**Marking procedure time (*n* of studies)**							
Mean procedural time ≤ 19 min	19	9	7	2	1	11	7
Mean procedural time > 19 min	15	4	7	2	2	8	7
**Complication (*n* of patients)**							
Pneumothorax (*n*)	117	49	57	11	–	115	2
Pulmonary haemorrhages (*n*)	59	37	18	4	–	56	3
Haemoptysis (*n*)	4	3	1	0	–	4	0
Other (*n*)	62	10	46	4	2	59	3
Successful marking (*n*, %)	3589 (95.9)	1246 (96.7)	1633 (97.6%)	474 (88.1)	236 (97.9)	2472 (97)	1086 (93.5)
**Failure analysis (*n*)**							
Total failure events	152	43	40	64	5	76	76
Insufficient injection	6	1	1	1	3	3	3
Leakage to the thoracic cavity	32	16	16	0	0	22	9
Inaccurate injection	14	10	4	0	0	4	7
Diffusion in the lung	6	0	5	0	1	2	3
Nodule characteristics–related failure	11	4	5	2	0	9	5
Other	8	0	5	3	0	3	5

### Methylene blue

Methylene blue (MB) has been used as the only fluorescent tracer in 1533 patients with 1673 nodules.

#### Dye characteristics

MB is a blue-colored thiazine dye. It can be administered orally, topically, or intravenously. MB has an excitation peak of approximately 670 nm and an emission peak at 690 nm. This range of fluorescence can interfere with the background autofluorescence emanated by haemoglobin and cytochromes. MB has a renal metabolism and is excreted through urine; thus, it should be avoided for renal insufficiency. Transient desaturation, cardiac arrhythmias, coronary vasoconstriction, reduced cardiac output, and decreased renal and mesenteric blood flow after intravenous administration have been recorded (13, 70, 71)

#### Administration modalities and dosages

Percutaneous CT-guided dye injection was used in two-thirds of the studies. The mean marking procedure time was reported in 14 papers. Luo et al. (24) reported the maximal marking procedure duration of 120 min.

The mean dosage of administered MB was 1.22 ml ranging from 0.04 to 7 ml. MB was administered in combination with autologous blood in two reports (19, 28), as a medical glue in two reports (33, 34), non-ionic contrast agent in two reports (15, 17), collagen in one report (31), and in a mixture of MB, fibrinogen, and thrombin in one report (24). No MB-related side effects were demonstrated in the different studies.

In most of the papers, the administration was performed the same day of the surgical procedure. In a report by Sun et al. (35), MB diffused on the pleural surface in a patient undergoing an 8-hour delay in surgery. Similarly, Zhang et al. (32) reported a localization failure in a patient undergoing resection 24 hours after the marking procedure. Vandoni et al. (17) found a significant correlation between the time interval between labelling and resection-affected MB density of coloration, resulting in three nodules undergoing localization failures at 190, 260, and 270 min. As shown in Figure 1, in most of the papers, MB was used for nodules ranging from 12 to 16 mm from the pleural surface, and the maximal depth was 60 mm (33).

#### Results

In the different studies, localization effectiveness ranged from 78.9 to 100%. In 8 of the 24 studies, all nodules were correctly resected after the MB marking procedure. Among the 1673 nodules, MB-guided surgery correctly localized and resected 1633 nodules (97.6% 95%CI [93.3%,98%]).

Marking procedure failures were recorded in detail in 14 reports. Leakage of the tracer to the pleural cavity due to pleural perforation or unprecise nodule puncturing was recorded in 16 marking procedures included in 10 different reports (17, 18, 21, 26, 28, 29, 33, 35, 36, 38). In three reports, including five localization failure events, MB diffused in the pulmonary parenchyma, resulting in a wide-labelled area including the nodule (19, 30, 38). Finally, four marking procedures failed due to misplaced trajectory during tracer injection (18, 36). Particularly, marking procedure–related failures were associated with excessive dilution of the tracer (28). In this paper, mixing MB with autologous blood helped to reduce color vanishing. Nodule characteristics–related failures were reported in seven reports (15, 17, 18, 26, 32, 36, 38). Particularly, extensive anthracosis, inadequate pulmonary exclusion during surgery, and subpleural nodules resulted in incorrect labelling procedures. Munoz et al. reported a marking failure in a 4 -mm nodule located 40 mm deep in the parenchyma (26). Finally, as previously stated, the interval time between the marking procedure and surgical resection significantly affected the labelling process (17, 32).

### Indocyanine green

ICG was used as the only fluorescent tracer in 1213 patients with 1289 nodules. In two-third of the studies, the mean nodule diameter was below 10 mm, and 50% of the nodules were GGO.

#### Dye characteristics

ICG is a hydro-soluble, anionic, and amphiphilic tricarbocyanine dye. It can be administered intravenously or topically. The excitation peak is at 780 nm and the emission peak is at 820 nm. This range of fluorescence prevents any interference with the background tissue autofluorescence. NIR fluorescence can vary in depths ranging from 0.5 to 1 cm. The safety spectrum is well established, with a low risk of adverse effects. ICG also allows multiple repeated uses due to its short half-life of 150 to 180 s and is cleared exclusively by the liver (6, 13).

#### Administration modalities and dosages

Percutaneous CT-guided dye injection was used in most of the studies. The mean marking procedure time was reported in 13 papers and ranged from 9 to 35 min. The marking procedure was accomplished in less than one hour.

Administered ICG solution concentration ranged from 0.125 mg/ml to 2.5 mg/ml. The total dosage of administered ICG ranged from 0.0125 mg to 1.25 mg of the dye. No ICG-related side effects were demonstrated in the different studies.

In most of the papers, the administration was performed the same day of the surgical procedure. Nonetheless, in 14 studies, the nodule marking procedure was accomplished preoperatively. In the report of Kim et al. (48), nodule marking was performed on an average 5 h before the procedure and up to 31 h before the procedure. The procedure still yielded successful intraoperative localization and resection of all the marked nodules with adequate oncological margins larger than the nodule diameter or larger than 20 millimeters, with the exception of one patient. In this case, margin infiltration was unrelated to nodule marking. In most of the papers, ICG was used for nodules ranging from 5 to 8 mm from the pleural surface, and maximal depth was 42 mm (48) (Figure 1).

#### Results

In the different studies, localization effectiveness ranged from 86 to 100%. In 8 of the 18 studies, all the nodules were correctly resected under ICG guidance. Among the 1289 nodules, ICG-guided surgery correctly localized and resected 1246 nodules (96.7% 95%CI [93.9%,98.4%]). Marking procedure failures were recorded in detail in seven reports. Particularly, marking procedure–related failures were associated with insufficient administration of ICG injection in 1 patient (44), and leakage of the tracer to the pleural cavity due to pleural perforation in 16 nodules was included in 5 different reports (44, 43, 49, 55, 46). Finally, inaccurate injection and failure of the bronchoscopic navigational system were reported by Geraci et al. (46) in seven and three cases, respectively. Nodule characteristics–related failures were reported in four studies (44, 39, 42, 55). Particularly, no pleural marks were developed in a nodule located at a 20 mm depth in the pulmonary parenchyma at a ICG dosage of 0,3ml of a 2,5 mg/ml ICG concentration (55). Anayama et al. (42), reported two failures for nodules located at 28 and 30 mm from the visceral pleura after endobronchial administration of ICG-iopamidol. Ujiie et al. (39) demonstrated no pleural marking of a centimetric nodule located at 48 mm from the visceral pleura. In another patient, the lung could not be properly deflated leading to fluorescent localization failure. Finally, Zhang et al. (44) failed to localize a 6 mm nodule located 1.1 mm from the pleura.

#### Surgical margins

Surgical margins were histologically negative because of neoplastic invasion in all nodules but two (41, 48). Surgical radicality was considered according to the guideline only in one study (48). In the other studies, surgical margins were considered adequate by the authors, but there is still no definition of surgical margin adequacy.

### Indigo carmine

Indigo Carmine has been used as the only dye fluorescent tracer in 442 patients with 583 nodules.

#### Administration modalities and dosages

Endobronchial dye injection was used in five out of eight enrolled reports. The mean marking procedure time ranged from 6 to 35 min (57, 58).

The administered IC dosage ranged from 0.5 to 2 ml, more frequently, 1 ml of dye tracer was injected. Dosages below 0.5 ml of IC are at high risk of localization failure (62). IC administration was performed up to 3 days before the surgical procedure (58). In a report by Hasegawa et al. (62), an interval time between marking and surgical procedure longer than 24 h significantly affected resection. In this report, a mixture of IC and a non-ionic contrast agent allowed intraoperative fluoroscopy, resulting in a complete resection of the nodules up to three days after the marking procedure. In most of the papers, IC was used for nodules from 5 to 17 mm from the pleural surface. Results: In the different studies, localization effectiveness ranged from 83.3 to 100%. In four of the eight studies, all the nodules were correctly resected after the IC marking procedure. Among the 538 nodules, IC-guided surgery correctly localized and resected 474 nodules (88.1% 95%CI [85.8%,99.3%]).

Marking procedure failures were recorded in detail in three reports (59, 57, 62).

Particularly, marking procedure–related failures were associated with an IC dosage below 0.5 ml and a three-day delay between labelling procedure and surgery (62). Nodule-related failures were associated with extensive anthracosis (57, 62) and parietal pleura to nodule distance >10 mm (59). Finally, Sato et al. demonstrated a 10% increase in failure risk in nodules requiring resection depth greater than 30 mm (59).

#### Surgical margins

Surgical margins were histologically negative because of neoplastic invasion in all the reports, with the exception of Sato et al. (59).

### Comparison between different fluorescent dye tracers

In this review, the efficacy of pulmonary nodule labelling for fluorescence-guided surgical resection of three different fluorescent dyes have been investigated. Methylene blue demonstrated the highest effectiveness in preoperative nodule labelling. Nonetheless, MB demonstrated a significant tendency to diffuse in the adjacent parenchyma. This result is consistent with previous findings (72). In order to avoid MB diffusion or dilution, surgery should be scheduled preferably the same day of nodule MB labelling due to the decrease of color intensity and the higher risk of targeting failure following the first 24 h from the targeting procedure (17, 32). Mixing MB with collagen or blood enhanced MB color intensity duration (19, 28, 31). ICG demonstrated similar results in terms of localization effectiveness. Nonetheless, the viscosity characterizing ICG and its ability to bind autologous collagen resulted in better results in terms of nodule definition and marking duration. Nonetheless, in Wen et al., the exceeding tracer could be removed by cleansing it with a surgical drape, following which the exact marking location was revealed (41). Kim et al. (48) demonstrated that ICG was still vividly detectable at surgery 3 days after the marking procedure. In a case report, ICG label could be detected up to 6 days after the marking procedure (73). IC showed the worst results in terms of nodule identification. This testifies to the limited number of reports on IC. Nonetheless, failure explanations were reported only in three of these papers for 5 failed procedures out of 64. Further studies are needed to evaluate IC in clinical practice, eventually building strategies to improve these results. In one of the reports (69), an endobronchial virtual-assisted-lung-mapping compared ICG and IC, demonstrating better results in the ICG group. The nodule depth from the pleural surface has a major impact on nodule localization effectiveness. As depicted in Figure 1, ICG demonstrated better results for subpleural nodules, while MB achieved better results in deeper nodules. IC was the tracer with a wider range of nodule-from-the-pleura deepness, but the overall number of studies are still limited.

### CT-guided procedure vs. endobronchial labelling procedure

Among the 55 reports included in this review, the fluorescent dye was injected through a CT-guided percutaneous administration in 30 of the enrolled studies, while endobronchial labelling of the nodules was used in 22 reports. From an analysis of data retrieved from 54 of the 55 papers, 2,324 patients underwent CT-guided labelling of 2548 nodules, while 1042 patients underwent bronchoscopic labelling of 1162 nodules. The rate of complications was higher in the CT-guided group, and pneumothorax was the most frequent complication experienced in this group. This is consistent with the finding of previous reports (42, 51). Thus, endobronchial localization can be particularly indicated for the emphysematous lung or for patients requiring labelling of multiple or bilateral nodules. The reasons underling localization failures in the reports included in the groups have been compared. The procedures failed due to leakage of the fluorescent dye to the thoracic cavity twice in the CT-guided group when compared with that in the endobronchial group, while inaccurate administration of injection was more frequent in the endobronchial group. As depicted in Figure 1, CT-guided labelling demonstrated better results for nodules up to 12 mm, while EBN labelling demonstrated better results in deeper nodules.

## Future research

With improvements in minimally invasive and robotic approaches, nodule localization strategies are gaining great importance. Therefore, future research studies are needed to address several unsolved issues. Particularly, comparative studies to evaluate the best fluorescence-based-tracer and to compare the best administration strategies are lacking in the different settings. Nonetheless, fluorescent tracers that can be absorbed by the tumors preserving fluorescence can lead future research.

## Conclusion

In this review, we analyzed the principal fluorescence-guided techniques used to visualize the lung nodule. Despite the accurate selection criteria, the heterogeneous nature of the studies could represent a limitation to identify the best technique. However, we can conclude that fluorescence-guided surgical resection of pulmonary nodules is safe and feasible. A preoperative marking procedure can facilitate surgical resection of hard-to-localize nodules. The choice between the tracer and the marking technique should be balanced by considering the nodule depth in the pulmonary parenchyma, tracer characteristics, and the time interval between marking procedure and surgery.
