# Supplementary figures and images for: Fluorescence-guided lung nodule identification during minimally invasive lung resections
Source: Front Surg. 2022 Jul 18;9:943829. doi: 10.3389/fsurg.2022.943829 (PMC9339676; doi:10.3389/fsurg.2022.943829)

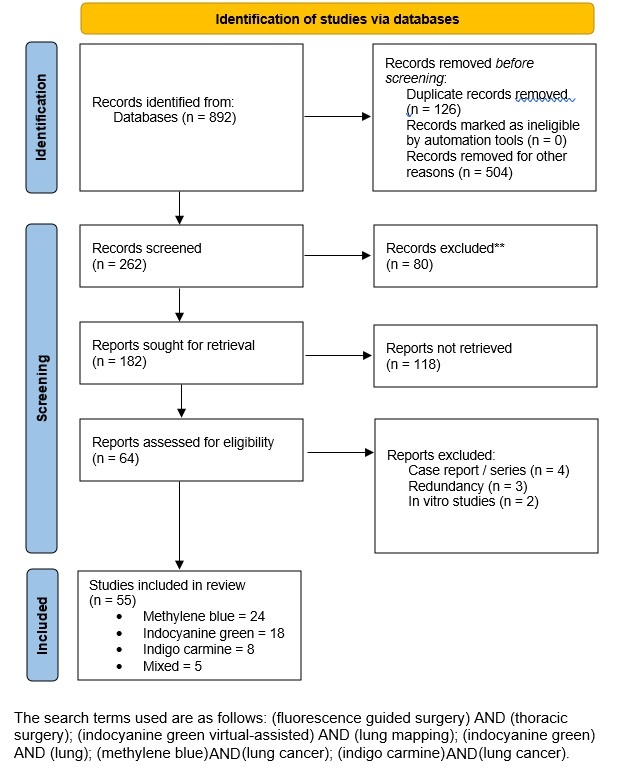

Supplement: Supplementary file 1 [file Image_1_v1.jpeg]
